# Enhancing CaV_0.5_Fe_0.5_O_3_-Based Lead-Free Perovskite Solar Cell Efficiency by over 23% via Transport Layer Engineering

**DOI:** 10.3390/nano15211646

**Published:** 2025-10-28

**Authors:** Syed Abdul Moiz, Muhammad I. Masud

**Affiliations:** 1 Device Simulation Laboratory, Department of Electrical Engineering, College of Engineering and Architecture, Umm Al-Qura University, Makkah 21955, Saudi Arabia; 2 Department of Electrical Engineering, College of Engineering, University of Business and Technology, Jeddah 21361, Saudi Arabia; m.masud@ubt.edu.sa

**Keywords:** solar cell, perovskite solar cell, lead-free, CaV_0.5_Fe_0.5_O_3_, CVFO, PEDOT:PSS, PCBM, TiO_2_, simulation, transport layer

## Abstract

In response to the rising global energy dilemma and associated environmental concerns, research into creating less hazardous solar technology has exploded. Due to their cost-effective fabrication process and exceptional optoelectronic properties, perovskite-based solar cells have emerged as promising candidates. However, their commercialization faces obstacles, including lead contamination, interface recombination, and instability. This study examines CaV_0.5_Fe_0.5_O_3_ (CVFO) as an alternative to lead-based perovskites, highlighting its improved stability and high efficiency through a series of simulation and modeling results. A record power conversion efficiency (PCE) of 23.28% was achieved (V_oc_ = 1.38 V, J_sc_ = 19.8 mA/cm^2^, FF = 85.2%) using a 550 nm thick CaV_0.5_Fe_0.5_O_3_ as an absorber. This was accomplished by optimizing the electron transport layer (ETL: TiO_2_, 40 nm, 10^20^ cm^−3^ doping) and the hole transport layer (HTL: Cu_2_O, 50 nm, 10^20^ cm^−3^ doping). Subsequently, it was established that defects at the ETL/perovskite interface significantly diminish performance relative to defects on the HTL side, and thermal stability assessments verified proper operation up to 350 K. To maintain efficiency, it is necessary to reduce series resistance (R_s_ < 1 Ω·cm^2^) and increase shunt resistance (R_sh_ > 10^4^ Ω·cm^2^). The findings indicate that CaV_0.5_Fe_0.5_O_3_ serves as a feasible alternative to perovskites and has the potential to enhance the performance of scalable solar cells.

## 1. Introduction

In the context of global energy crisis and growing awareness of climate change, researchers have progressively campaigned for more efficient and environmentally sustainable solar power instead of conventional solar energy technology [1,2,3,4,5]. Recently discovered organic–inorganic metal halide perovskites have significantly impacted solar cell technology due to their exceptional properties, including inexpensive manufacturing costs, extended charge mobility, high light absorption, and adjustable energy levels. Since its inception in 2009, perovskite solar cells have seen significant advancements, with power conversion efficiency increasing from 3.8% to over 29%, positioning them as a credible alternative to conventional silicon solar systems [6,7].

Among huge number of perovskites, lead-based perovskites show high photovoltaic efficiency; however, these perovskites are severely hampered by the issue of lead-based toxicity. This poisoning results in soil and water contamination, subsequently leading to cardiovascular diseases, some abnormal tissue growth, and neurological disorders in humans and animals [8,9,10]. Research on lead-free perovskite absorbers, including those based on tin, germanium, bismuth, or double perovskite (A_2_BB’X_6_) materials, has increased due to concerns regarding lead-based toxicity [11,12,13]. These materials aim to preserve advantageous optoelectronic properties while eliminating lead metal. Unlike lead halide perovskites, these novel absorbers exhibit several limitations that hinder their practical application. Factors include low power conversion efficiency, elevated defect concentrations, inadequate stability, and rapid oxidation (e.g., Sn^3+^ to Sn^4+^). A significant challenge in advancing sustainable perovskite solar cell technology is the absence of a candidate that fully replicates the performance of lead-based systems. The ongoing exploration of alternative perovskites emphasizes the need to balance non-toxicity, long-term stability, and high efficiency [14].

Present approaches for reducing lead toxicity in perovskite solar cells, like encapsulation to stop lead leakage or partial replacement with Sn^2+^ and Ge^2+^, have not worked very well. The challenge of creating an encapsulation with a thin layer is the difficult-to-control penetration that depends on transport via localized pinholes. To ensure that electronics remain stable and dependable, new encapsulating materials and technologies are needed to meet the encapsulation criteria and stop oxygen and moisture from penetrating [15]. On the other hand, environmental variables, manufacturing techniques, and materials significantly influence the encapsulation of perovskite solar cells. Increased processing temperatures, combined with moisture and oxygen ingress from the encapsulant, might compromise the overall integrity of the perovskite layer [16].

Among several lead-free perovskites, CaV_0.5_Fe_0.5_O_t_ stands out as a possible substitute absorber due to its substantial light absorption and suitable bandgap of around 1.8 eV. Improved thermal and chemical robustness provides longevity under operating settings for CaV_0.5_Fe_0.5_O_3_, in contrast to Pb-based systems. Importantly, CaV_0.5_Fe_0.5_O_3_ can be scaled up for large-scale deployment and is made of earth-abundant, non-toxic materials, which solves environmental problems. With its impressive efficiency, stability, and sustainability, CaV_0.5_Fe_0.5_O_3_ stands out as a potential contender in the continuous quest for next-generation perovskite materials that may surpass the constraints of present lead-based technology [17,18,19].

The challenges associated with CaV_0.5_Fe_0.5_O_3_ as a perovskite material stem from its lowered efficiency relatively compared to other lead-based perovskite solar cells, primarily due to insufficient charge transport characteristics and optimization. While Pb-based perovskites such as MAPbI_3_ exhibit excellent charge carrier mobility and lower recombination rates, CaV_0.5_Fe_0.5_O_3_ also faces challenges due to poor charge extraction and sensitive charge trapping, limiting its effectiveness in solar cell applications. To enhance efficiency, it is essential to optimize the electrical structure of the proposed photovoltaic devices, reduce defect densities, and facilitate interfacial charge transfer through compositional tuning and doping [20,21]. Addressing these challenges could position CaV_0.5_Fe_0.5_O_3_ as a more viable and environmentally friendly alternative to harmful lead-based perovskites in solar cell applications [18,22].

A significant disadvantage of some oxide perovskites is their typically higher exciton binding energy (EB), which hinders the dissociation of photogenerated excitons into free carriers at ambient temperature. Consequently, our simulations provide a potential peak performance, based on optimal free carrier generation. Low practical open-circuit voltage and short-circuit current are anticipated due to significant geminate recombination losses resulting from a high EB. Therefore, more experimental efforts must achieve a low EB by altering the composition or exploring alternative methods to enhance exciton dissociation in this material if the asserted high efficiency is to be credible.

Like other types of solar cells, the performance of CaV_0.5_Fe_0.5_O_3_ perovskite solar cells is also dependent upon efficient charge extraction via suitably aligned hole and electron transport layers (HTLs/ETLs). This study proposes that efficient electron transport in CaV_0.5_Fe_0.5_O_3_-based solar cells can be achieved by employing three potential electron transport layers: TiO_2_, SnO_2_, and ZnO. TiO_2_ demonstrates significant chemical stability and appropriate band alignment, facilitating effective electron extraction from the perovskite layer [23]. SnO_2_ exhibits high electron mobility and facilitates low-temperature processing, thereby improving charge collection and allowing for the fabrication of flexible devices [24]. ZnO demonstrates enhanced conductivity and rapid electron transport; however, its interfacial reactivity with perovskites necessitates surface passivation to mitigate degradation [25]. Each ETL can be optimized to enhance charge extraction, reduce recombination, and improve the overall efficiency of CaV_0.5_Fe_0.5_O_3_ solar cells.

For optimal hole extraction from CaV_0.5_Fe_0.5_O_3_ perovskite solar cells, three established hole transport materials—PEDOT:PSS, Spiro-OMeTAD, and Cu_2_O—were chosen based on criteria such as band alignment, stability, and charge mobility. PEDOT:PSS demonstrates high conductivity and favorable solution processability; nonetheless, it is susceptible to degradation in acidic environments [26,27]. Spiro-OMeTAD demonstrates enhanced film homogeneity and aligned energy levels; however, it requires dopants to enhance conductivity. Cu_2_O is recognized as the most effective hole transport layer due to its high hole mobility (~80 cm^2^/V·s), favorable valence band alignment with CaV_0.5_Fe_0.5_O_3_, and exceptional chemical stability [28]. These characteristics make hole extraction more effective and increase the long-term robustness of high-performance lead-free photovoltaic systems.

This research aims to develop lead-free perovskite solar cells by replacing toxic lead (Pb) with the environmentally friendly and stable absorber layer CaV_0.5_Fe_0.5_O_3_. As a result, both thermal stability and power conversion efficiency will be enhanced. The study improves charge transport layers in CaV_0.5_Fe_0.5_O_3_-based perovskite solar cells through the optimization of thickness and doping density, which aims to reduce recombination losses and enhance charge extraction. This is accomplished by selecting appropriate electron transport layers (TiO_2_, SnO_2_, ZnO) and hole transport layers (PEDOT:PSS, Spiro-OMeTAD, Cu_2_O). This study also optimizes the thickness of the CaV_0.5_Fe_0.5_O_3_ absorber layer to balance charge transport and light absorption, evaluates thermal stability through temperature effects, and analyses series and shunt resistances to improve device performance. The photovoltaic parameters (V_oc_, J_sc_, FF, and PCE) are determined using SCAPS-1D numerical modeling, and the device design (ITO/ETL/CAV_0.5_FE_0.5_O_3_/HTL) is verified using quantum and photovoltaic efficiencies. The key novel aspects of this study are listed here:

(i) Novel study: First thorough study of CaV_0.5_Fe_0.5_O_3_ as a novel perovskite solar cell material.
(ii) Material integration innovation: CaV_0.5_Fe_0.5_O_3_ is abundant and non-toxic, with enhanced TiO_2_/Cu_2_O charge transport layers.
(iii) Efficiency Record: Lead-free perovskite-inspired solar cells converted 23.28% electricity.
(iv) Exceptional stability: Showed thermal stability of up to 100 °C.
(v) Ideal band alignment: Ensured energy-level correlation between transport layers and CaV_0.5_Fe_0.5_O_3_ (~3.7 eV conduction band minimum/~5.5 eV valence band maximum).

## 2. Materials and Methods

### 2.1. Device Inverted Configuration

Both n-i-p (inverted) and p-i-n (non-inverted) perovskite solar cell topologies have exhibited unprecedented power conversion efficiency, highlighting their feasibility. This study concentrates on optimizing an n-i-p structure due to its proven charge transport dynamics and substantial experimental dataset for model validation, while acknowledging the significant recent advancements in inverted devices. Inverted structures have recently achieved confirmed efficiencies of up to 25.7%, representing a groundbreaking advancement for perovskite solar cells [29]. This also validates the n-i-p design as a premier platform for high-performance and robust photovoltaics, so our modeling framework ITO/ETL/CAV_0.5_FE_0.5_O_3_/HTL aims to enhance this promising configuration.

### 2.2. Simulation Software

The computational modeling of the proposed lead-free perovskite solar cell was conducted using SCAPS-1D (version 3.3.10), a recognized software tool for photovoltaic device modeling [30]. SCAPS-1D serves as a coupled-differential equation solver that self-consistently computes charge transport, recombination dynamics, and optical-electronic coupling by solving the Poisson equation and the continuity equations for electrons and holes under steady-state conditions [30,31,32]. The software integrates validated physical models, including Shockley–Read–Hall (SRH) recombination, band-to-band tunneling, and defect-assisted tunneling, enabling accurate predictions of key performance metrics such as J–V curves, EQE, and temperature-dependent efficiency for layered solar cell architectures. Its proven reliability in simulating perovskite [33], polymer [34], CIGS [35], and heterojunction solar cells [36] makes it particularly suitable for optimizing the proposed CaV_0.5_Fe_0.5_O_3_-based device structure.

### 2.3. Simulation Device Models

A collection of fundamental mathematical models is used to simulate the photovoltaic response in SCAPS-1D:

(1) In electrostatics, the Poisson equation governs, which can be written as [37,38,39,40,41]

(1) $$\frac{d^{2}\phi \left(x\right)}{dx^{2}}=\frac{e}{\epsilon_{o}\epsilon_{r}}\left(p\left(x\right)-n\left(x\right)+N_{D}-N_{A}+\rho_{p}-\rho_{n}\right)$$

Here, the Poisson model makes use of standardized notation for its key parameters. The electric charge is represented by “e”, which is equal to 1.602 × 10^−19^ C. Both absolute (ϵ_0_ = 8.85 × 10^−12^ F/m) and relative (ϵᵣ) dielectric constants are used. Deep donors (N_D_) and shallow acceptors (N_A_) are the doping densities. The free hole density (ρ_p_) and the free electron density (ρ_n_) are the carrier distributions. The spatial profiles of holes are denoted as p(x) and electrons as n(x).

(2) Electron and hole continuity equations define charge carrier dynamics, which can be defined as [37,38,39,40,41]

(2) $$\frac{dJ_{n}}{dx}=G-R$$

(3) $$\frac{dJ_{p}}{dx}=G-R$$

Here, the continuity equations quantitatively define the correlation between the spatial gradients of the electron and hole current densities, J_n_(x) and J_p_(x), and the local net generation rate of free carriers, defined as the gap between the generation (G) and recombination (R) processes.

(3) Equations for total charge, electron and hole specific charges, and charge transport describe this phenomenon, and can be modeled as [37,38,39,40,41]

(4) $$J=J_{n}+J_{p}$$

(5) $$\;J_{n}=D_{n}\frac{dn}{dx}+\mu_{n}n\frac{d\phi }{dx}$$

(6) $$J_{p}={-D}_{p}\frac{dp}{dx}+\mu_{p}p\frac{d\phi }{dx}$$

The charge transport mechanics of a p-i-n junction in the modeling environment is directly comparable to those of a solar cell. The chosen basic model disaggregates the entire current density into its component parts: hole (J_p_) and electron (J_n_) contributions. Each component is further obtained from the aggregate of drift (proportional to carrier mobilities (µ_p_/µ_n_)) and diffusion (which is proportional to the diffusion coefficients D_p_, D_n_) currents.

(4) The optical absorption coefficient equation is used to model the interaction with light. For the optical absorption model, we choose the established classic model from SCAPS-1D’s selection. Here we can define the optical absorption coefficient “α” as α(λ) in the conventional paradigm, where “λ” is the optical wavelength and “hυ” is the energy. “Eg” represents the energy bandgap of the associated thin-film layer, while “A” and “B” are specified as arbitrary constants in this model according to Equation (7). The classic optical model is expressed as [37,38,39,40,41]

(7) $$\alpha \;(\lambda )=\left(A+\frac{B}{h\nu }\right)\sqrt{h\nu -E_{g}}$$

With this set of these equations, we may numerically solve for the performance of solar cells in one dimension. Under the AM. 1.5 typical illumination condition, all the simulations were performed through SCAPS-1D at a temperature of 300 K.

### 2.4. Energy Level Band Alignment

**Figure 1:**
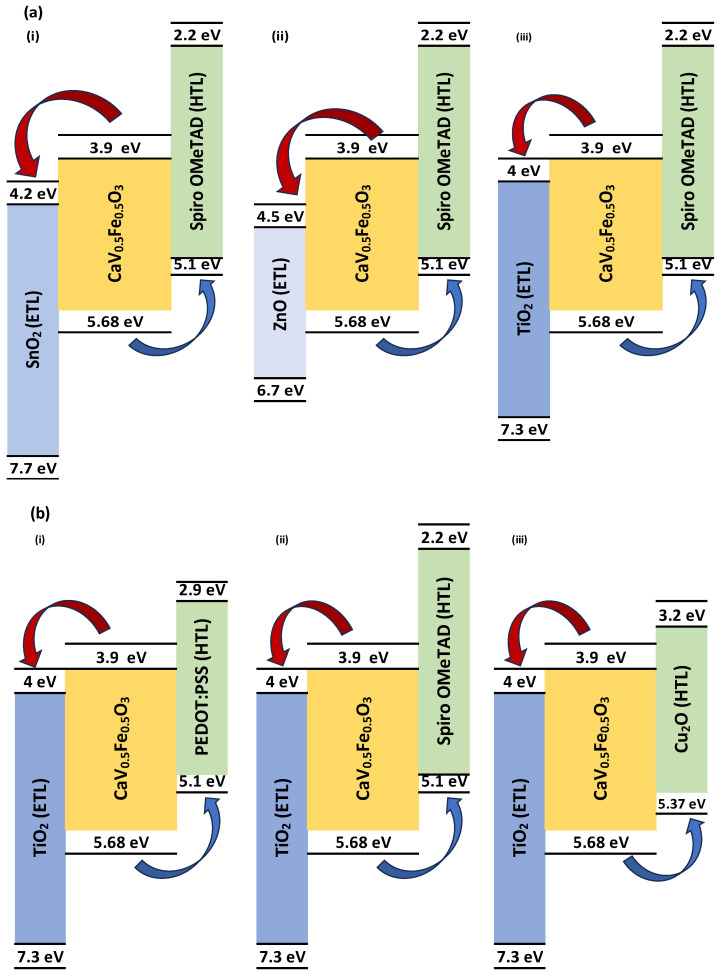
Energy level graphs illustrating the band alignment between a CaV_0.5_Fe_0.5_O_3_ perovskite absorber (VBM: −5.68 eV, CBM: −4.2 eV) and many charge transport layers. (**a**) The electron transport layer (ETL), alongside the hole transport layer (HTL): Spiro OMeTAD is constant, while the ideal ETL is determined by varying (i) SnO_2_, (ii) ZnO, (iii) TiO_2_. Likewise, (**b**) the hole transport layer (HTL), with the fixed optimum electron transport layer (ETL), Cu_2_O, determines the optimal ETL by varying (i) PEDOT:PSS, (ii) Spiro OMeTAD, (iii) Cu_2_O.

The energy level alignment diagram as shown in Figure 1 illustrates that the chosen hole and electron transport materials meet the essential criteria for effective charge extraction in a solar cell. Each transport layer demonstrates suitable band energies in relation to the CaV_0.5_Fe_0.5_O_3_ absorber, enabling the directed flow of holes towards the cathode and electrons towards the anode. This essential alignment reduces energy barriers at the interfaces, consequently reducing charge recombination losses and facilitating high-performance device operation [42,43].

### 2.5. Device Simulation Parameters

Both Table 1 and Table 2 outline the material parameters requisite for SCAPS-1D modeling, including the electric, optical, and other physical parameters including defect density for the electronic transport layer and hole transport layer, respectively, that govern charge carrier dynamics in a perovskite solar cell system, respectively. The information in Table 1 and Table 2 was derived from a combination of experimental and theoretical studies, as referenced in the citations. Due to the novelty of CaV_0.5_Fe_0.5_O_3_, the majority of the data was derived from theoretical research [44,45,46,47,48,49,50,51,52,53,54,55,56,57,58,59,60,61,62,63,64]. The energy band gap (E_g_), electron affinity (χ), dielectric permittivity (ε), effective density of states in the conduction and valence bands (Nc, Nv), carrier mobilities (µ_e_, µ_h_), and doping concentrations (Nd, Na) are critical parameters for each layer, including the electron transport layers (ETLs: SnO_2_, ZnO, TiO_2_), the perovskite absorber (CaV_0.5_Fe_0.5_O_3_), and the hole transport layers (HTLs: PEDOT:PSS, Spiro-OMeTAD, Cu_2_O). The alignment of bands, charge generation, charge transport, recombination rates, and overall carrier extraction efficiency are all influenced by these factors. Collectively, these characteristics create the foundation for predicting and improving the performance of perovskite solar cells across various transport layer configurations.

### 2.6. Simulation Flow Chart

To determine the charge transport layer and optimal design that may yield the maximum power conversion efficiency, it is essential to execute the subsequent simulation phases on the devices using the proposed CaV_0.5_Fe_0.5_O_3_-based perovskite solar cell.

Phase 1: Define all necessary simulation settings for each device layer as per the requirements of Table 1 and Table 2, respectively.
Phase 2: Establish the physical and material properties of the perovskite absorber layer, the hole transport layer, and the electron transport layer by consulting the literature, as detailed in Table 1 and Table 2, respectively [18,44,45,46,47,48,49,50,51,52,53,54,55,56,57,58,59,60,61,62,63].
Phase 3: Recommend (initial guess) the thickness range and doping density for each layer for initialization of the simulation.
Phase 4: Optimization of ETL Thickness: Determine the ideal thickness of ETL (SnO_2_, ZnO, and TiO_2_) for each device with a fixed HTL (such as spiro OMeTAD as the hole transport layer), using a series of simulations that provide the greatest power conversion efficiency. Subsequently, update the running parameters of simulations using the ideal thickness of each ETL for further simulations.
Phase 5: Evaluation of photovoltaic characteristics in relation to varying ETL thicknesses: assess the photovoltaic metrics, including open-circuit voltage, short-circuit current, fill factor, and power conversion efficiency of each device as a function of each ETL.
Phase 6: Evaluation of photovoltaic characteristics in relation to varying ETL doping density: assess the photovoltaic metrics, including open-circuit voltage, short-circuit current, fill factor, and power conversion efficiency of each device as a function of each ETL’s doping density.
Phase 7: Identify the most efficient electron transport layer among SnO_2_, ZnO, and TiO_2_ via the evaluation of power conversion efficiency, and thereafter use the optimal electron transport layer, together with its ideal thickness and doping density, for further optimization of the hole transport layer.
Phase 8: Optimization of HTL Thickness: Determine the ideal thickness of HTL (PEDOT:PSS, Spiro OMeTAD, Cu_2_O) for each device with a fixed ETL (optimum electron transport layer determined from step 7), using a series of simulations that provide the greatest power conversion efficiency. Subsequently, update the running parameters of simulations using the ideal thickness of each HTL for further simulations.
Phase 9: Evaluation of photovoltaic characteristics in relation to varying HTL thicknesses: Assess the photovoltaic metrics, including open-circuit voltage, short-circuit current, fill factor, and power conversion efficiency of each device as a function of each HTL.
Phase 10: Evaluation of photovoltaic characteristics in relation to varying HTL doping density: assess the photovoltaic metrics, including open-circuit voltage, short-circuit current, fill factor, and power conversion efficiency of each device as a function of each HTL’s doping density.
Phase 11: Identify the most efficient hole transport layer among S PEDOT:PSS, Spiro OMeTAD, and Cu_2_O via the evaluation of power conversion efficiency, and thereafter use the optimal HTL, together with its ideal thickness and doping density, for further optimization of the absorber layer.
Phase 12: Optimization of absorber layer (CaV_0.5_Fe_0.5_O_3_) thickness: determine the ideal thickness of for the absorber layer where both ETL and HTL are the most efficient transport layers as determined in phase 7 and phase 11, respectively.
Phase 13: Simulate the current-voltage (J–V) characteristics of the optimized electron transport layer, hole transport layer, and absorber CaV_0.5_Fe_0.5_O_3_ layer under AM1.5G illumination to extract essential photovoltaic parameters: short-circuit current density, open-circuit voltage, fill factor, and maximum possible power conversion efficiency.
Phase 14: Assess the quantum efficiency (QE) spectra for both the original unoptimized and fully optimized device configurations to measure performance improvement across the absorption spectrum.
Phase 15: Examine the influence of interface defect (or trap) density at the Perovskite/ETL and Perovskite/HTL interfaces on the performance metrics (V_oc_, J_sc_, FF, PCE) of a highly optimized device.
Phase 16: Assess the temperature-dependent photovoltaic efficacy of the highly optimized device throughout a spectrum of operating temperatures (up to 400 K).
Phase 17: Examine the impact of series (R_S_) and shunt (R_SH_) resistances on the photovoltaic performance of the highly optimized device.
Phase 18: End of simulation.

## 3. Results and Discussion

### 3.1. Thickness Optimization of Electron Transport Layer

**Figure 2:**
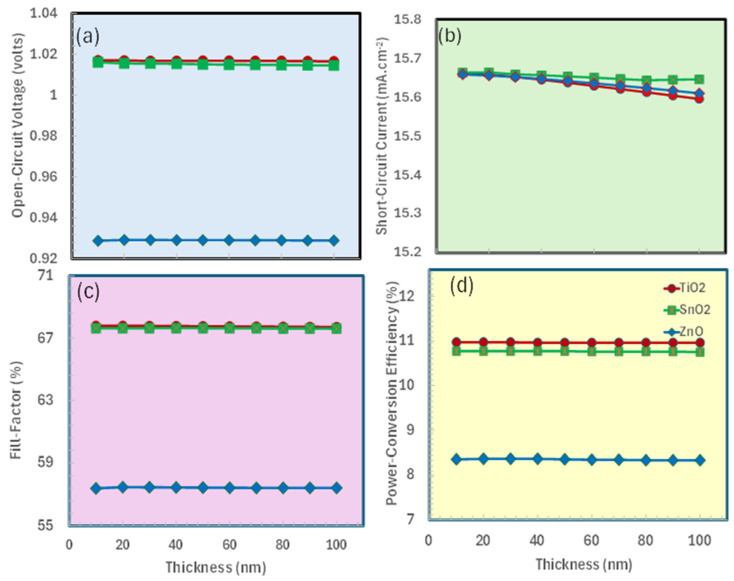
Photovoltaic performance parameters of ITO/ETL/CaV_0.5_Fe_0.5_O_3_/Spiro OMeTAD as a function of electron transport layer thickness: (**a**) open-circuit voltage (V_oc_), (**b**) short-circuit current density (J_sc_), (**c**) fill factor (FF), (**d**) power conversion efficiency (PCE).

The photovoltaic response of the ITO/ETL/CaV_0.5_F_0.5_O_3_/Spiro-OMeTAD solar cell structure was thoroughly investigated concerning the ETL thickness for three widely accepted electron transport materials: TiO_2_, SnO_2_, and ZnO, as shown in Figure 2. We originally picked spiro-OMeTAD as a typical hole transport layer material to determine the appropriate ETL parameters, owing to its widespread use in perovskite solar cells. This random selection enabled us to isolate and assess the ETL’s performance separately prior to proceeding with comprehensive HTL optimization.

The thickness of the ETL is crucial for optical management, as it must be sufficiently thin to reduce parasitic absorption while still being thick enough to guarantee effective charge extraction [64,65]. The analysis determined specific thicknesses for each ETL, where the power conversion efficiency reached its maximum: 40 nm for TiO_2_, 65 nm for SnO_2_, and 60 nm for ZnO, see Figure 2d. These optimum values signify a trade-off between optical transparency and charge transfer efficacy. Thin ETLs may encounter insufficient charge extraction, but thicker layers might lead to high resistance and diminish light absorption in the perovskite layer. The production of photocurrent is enhanced by the appropriate adjustment of the ETL thickness, which improves light coupling into the absorber. TiO_2_ likely demonstrated enhanced performance at its optimum thickness because of its elevated electron mobility and advantageous band alignment with CaV_0.5_F_0.5_O_3_, which is reasonably significant. These results highlight the importance of optimizing the thickness of the electron transport layer in perovskite solar cells.

### 3.2. Doping-Density Optimization of Electron Transport Layer

The efficiency of perovskite solar cells based on CaV_0.5_Fe_0.5_O_3_ is greatly affected by the doping density of the light-facing electron transport layer, which in turn affects charge extraction, recombination kinetics, and optical characteristics. The ideal doping density reduces interfacial recombination and increases conductivity and electric field strength for effective electron collecting. In contrast, a decrease in open-circuit voltage and a rise in parasitic absorption and a reduction in short-circuit current are caused by defect-mediated recombination centers introduced by excessive doping. Therefore, an optimized doped ETL balances these trade-offs for highly efficient perovskite solar cell [66,67].

**Figure 3:**
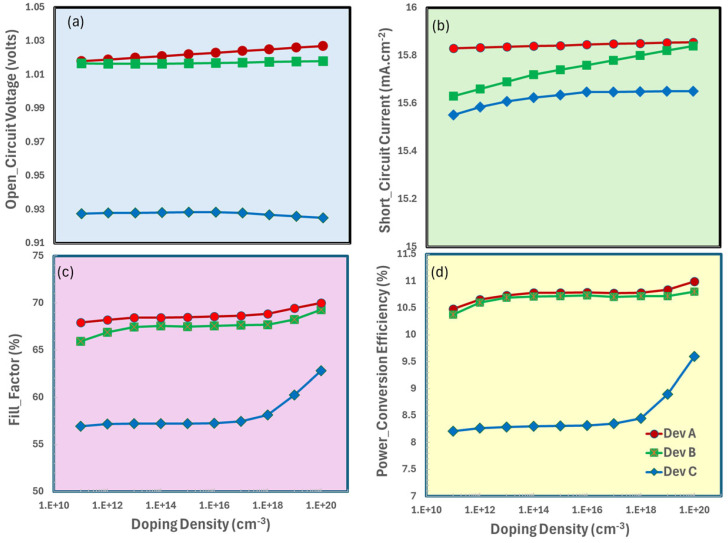
Photovoltaic performance parameters of ITO/ETL/CaV_0.5_Fe_0.5_O_3_/Spiro OMeTAD as a function of electron transport layer (ETL) doping-density: (**a**) open-circuit voltage (V_oc_), (**b**) short-circuit current density (J_sc_), (**c**) fill factor (FF), (**d**) power conversion efficiency (PCE).

The device’s ITO/ETL/CaV_0.5_Fe_0.5_O_3_/Spiro OMeTAD (where ETL = {TiO_2_, SnO_2_, ZnO}) exhibits optimum photovoltaic performance at a doping density of 10^20^ cm^−3^, as seen in Figure 3, with the open-circuit voltage (Figure 3a) attaining significant peaks owing to substantially reduced charge recombination at this optimal doping level. Concurrently, the short-circuit, current density (Figure 3b) reaches its maximum value, indicating effective charge collection facilitated by enhanced conductivity and carrier mobility at this doping concentration. As series resistance decreases, the fill factor (Figure 3c) exhibits a progressive enhancement of up to 10^20^ cm^−3^, after which it stabilizes as more doping provides diminishing returns. The power conversion efficiency (Figure 3d) finally embodies these integrated trends, reaching its maximum value at this critical doping concentration when V_oc_, J_sc_, and FF are all optimal. This underscores the importance of 10^20^ cm^−3^ as the optimal doping concentration for improving the device’s overall performance.

After doping optimization, it is observed that TiO_2_ exhibited the highest power conversion efficiency compared to the other evaluated ETL materials (SnO_2_, ZnO) for CaV_0.5_Fe_0.5_O_3_-based perovskite solar cells. The TiO_2_ managed to effectively extract electrons while reducing recombination losses due to its optimal band alignment (~3.5 eV bandgap, 3.8 eV electron affinity) with the CaV_0.5_Fe_0.5_O_3_ absorber. The perovskite layer’s light utilization was improved by the chemically stable and broad bandgap properties of TiO_2_, which decreased parasitic absorption. These results verify that TiO_2_ is the optimal electron transport layer for this system. Now we will apply TiO_2_ as ETL to determine the optimal hole transport layer configuration, which provides balanced charge transfer and enhances the device’s performance.

### 3.3. Thickness Optimization of Hole Transport Layer

To achieve high-performance perovskite solar cells, optimizing the hole transport layer is crucial [68]. HTL governs hole extraction efficiency, minimizes interfacial recombination, and ensures steady energy band alignment with the perovskite absorber. This work utilized PEDOT:PSS, Spiro OMeTAD, and Cu_2_O as the hole transport layer as discussed above. PEDOT:PSS is a prominent hole transport material that provides excellent conductivity and solution processability. Spiro-OMeTAD, conversely, offers superior film homogeneity and aligned energy levels. Conversely, Cu_2_O is a viable inorganic alternative that exhibits enhanced chemical stability, increased hole mobility (~80 cm^2^/V·s), and optimal valence band alignment (~5.3 eV) with CaV_0.5_Fe_0.5_O_3_.

**Figure 4:**
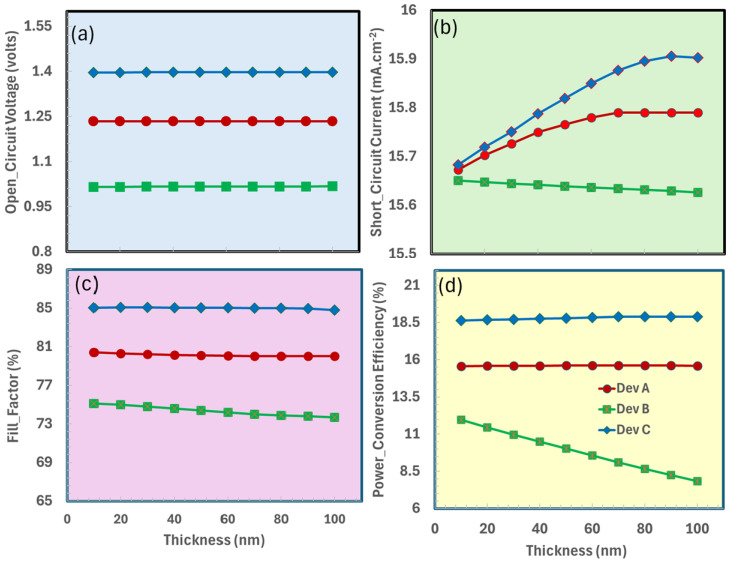
Photovoltaic performance parameters of the devices ITO/TiO_2_/CaV_0.5_Fe_0.5_O_3_/HTL as functions of hole transport layer thickness: (**a**) open-circuit voltage (V_oc_), (**b**) short-circuit current density (J_sc_), (**c**) fill factor (FF), (**d**) power conversion efficiency (PCE).

Figure 4 depicts the optimization of the hole transport layer thickness for ITO/TiO_2_/CaV_0.5_Fe_0.5_O_3_/HTL, where HTL comprises {Dev A (PEDOT:PSS), Dev B (Spiro OMeTAD), and Dev C (Cu_2_O)}, and its impact on essential photovoltaic parameters. In Figure 4a, the open-circuit voltage demonstrates a non-linear correlation with HTL thickness, attaining its zenith at an optimal thickness where charge transport and light absorption are ideally coordinated, peaking at intermediate values due to balanced charge extraction and recombination. Figure 4b illustrates that the short-circuit current progressively diminishes with increasing HTL thickness, indicating heightened parasitic absorption in thicker layers (Figure 4c). The power conversion efficiency has a similar trend to the open-circuit voltage (V_oc_), reaching its peak at an appropriate hole transport layer thickness where charge transfer and light absorption are ideally matched. The evaluated HTL materials—PEDOT:PSS, Spiro-OMeTAD, and Cu_2_O—exhibit divergent performance patterns. Cu_2_O demonstrates enhanced stability and efficiency at lower densities, which is attributable to its improved band alignment with the perovskite absorber and elevated hole mobility.

### 3.4. Doping-Density Optimization of Hole Transport Layer

Doping the hole transport layer is crucial for the proper operation of perovskite solar cells, as it enhances the layer’s capacity to collect and transport holes from the perovskite to the electrode. The conductivity of the HTL may be enhanced by precisely modifying the doping density, hence minimizing energy losses and increasing the device’s efficiency. Excessive doping may result in the defects formation that trap charges and reduce performance, while inadequate doping could affect hole mobility. Reducing recombination losses and facilitating an efficient charge transport can be achieved through the optimal doping density of the hole transport layer, thereby improving the overall efficiency of the solar cell [69].

**Figure 5:**
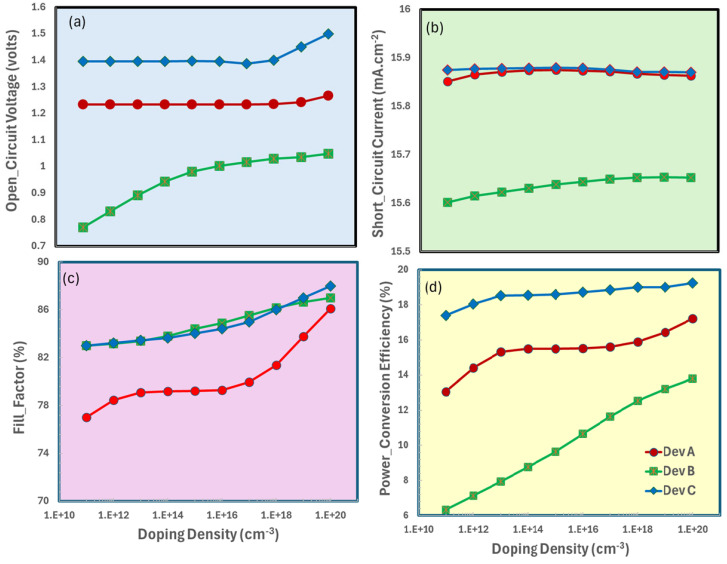
Photovoltaic performance parameters of the devices ITO/TiO_2_/CaV_0.5_Fe_0.5_O_3_/HTL as a function of hole transport layer doping-density: (**a**) open-circuit voltage (V_oc_), (**b**) short-circuit current density (J_sc_), (**c**) fill factor (FF), (**d**) power conversion efficiency (PCE).

Figure 5 depicts the influence of doping density (ranging from 10^10^ to 10^20^ cm^−3^) of the hole transport layer on the photovoltaic characteristics of perovskite solar cells, demonstrating that device performance is significantly impacted by optimal doping levels. The photovoltaic trends are roughly same for all HTL-based solar cells. The open-circuit voltage initially increases with doping, reaching its maximum at moderate levels because of enhanced charge extraction and reduced recombination. However, it quickly decreases at high doping concentrations; defect-mediated recombination may become dominant. Likewise, the short-circuit current remains rather steady throughout moderate doping levels, but it experiences a little decrease at high doping concentrations owing to increased parasitic absorption and series resistance. In the optimal doping zone, when charge transport is balanced and resistive losses are minimized, the fill factor reaches its maximum, mirroring the trend of V_oc_. The power conversion efficiency ultimately reflects both trends, achieving optimum performance at intermediate doping levels (10^19^–10^20^ cm^−3^), when the advantages of improved conductivity and charge collection outweigh the detrimental consequences of defect formation. This demonstrates the critical need for careful doping adjustment to improve solar cell efficiency while preserving steady performance.

### 3.5. Thickness Optimization of Absorber Layer

Optimizing the thickness of the CaV_0.5_Fe_0.5_O_3_ perovskite absorber layer is crucial for improving the power conversion efficiency of perovskite solar cells. To effectively minimize charge recombination losses, it is essential to determine an optimal thickness that ensures adequate light absorption. The photocurrent could decrease if the layer is excessively thin, as it may not be able to absorb enough photons. A thick layer can increase bulk resistance and restrict charge transfer, resulting in a decrease in both the fill factor and open-circuit voltage. Furthermore, an optimal thickness improves the device’s overall performance by reducing defect-related recombination losses and enhancing charge carrier extraction [18,54,70,71,72,73].

**Figure 6:**
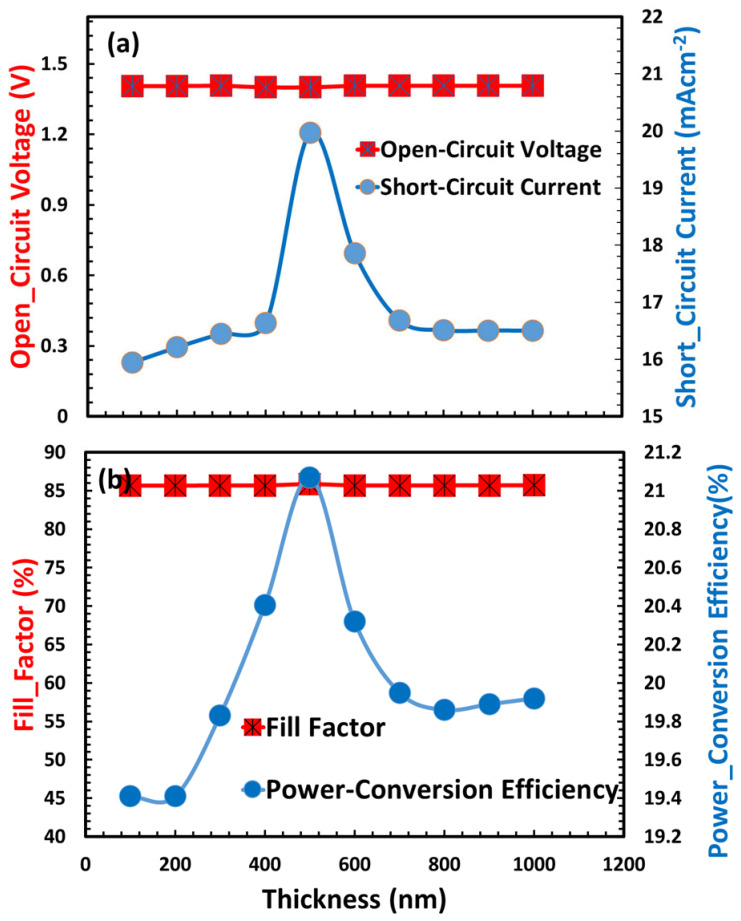
Photovoltaic performance parameters of ITO/TiO_2_/CaV_0.5_Fe_0.5_O_3_/Spiro OMeTAD as a function of perovskite absorber layer (CaV_0.5_Fe_0.5_O_3_) thickness.

Figure 6a,b shows the correlation between the perovskite absorber layer thickness and photovoltaic parameters, which clearly demonstrate a balance between charge transport and photon absorption. At first, the short-circuit current (see Figure 6a) rises with the thickness of CaV_0.5_Fe_0.5_O_3_ absorber layer because of improved photon absorption; however, it reaches a saturation point and starts to decrease as bulk recombination and parasitic losses become more significant. The open-circuit voltage (see Figure 6a) exhibits a gradual decline with increasing thickness, attributed to elevated recombination rates that diminish quasi-Fermi level splitting. Concurrently, the fill factor (see Figure 6b) experiences a reduction at larger thicknesses, primarily due to heightened series resistance and suboptimal charge extraction. The power-conversion efficiency reaches its maximum of 23.28% at an intermediate thickness of approximately 550 nm. This thickness represents a balance between optimal light harvesting and minimal recombination losses, underscoring the importance of thickness optimization in enhancing solar cell performance by aligning the absorption depth with the charge diffusion length.

### 3.6. Final Optimized Device Parameters

Table 3 outlines the optimal thickness and doping density for each layer to enhance the photovoltaic response of the proposed perovskite solar cell. The electron transport layer is composed of a 40 nm thick TiO_2_ with a doping density of 1.0 × 10^20^ cm^−3^. The 50 nm thick Cu_2_O functions as the hole transport layer with a corresponding doping density. The thickness of the CaV_0.5_Fe_0.5_O_3_ layer is 550 nanometers. The proposed device achieved a power conversion efficiency of 23.28%, characterized by an open-circuit voltage of 1.38 V, a short-circuit current density of 19.8 mA/cm^2^, and a fill factor of 85.2%, following the designated optimization process. The enhanced PCE results from the thoroughly engineered ETL and HTL, which effectively extract and transfer charges. The properties of the absorber layer can be optimized to improve performance. 

### 3.7. Optimized Photovoltaic and Quantum Efficiency Responses

**Figure 7:**
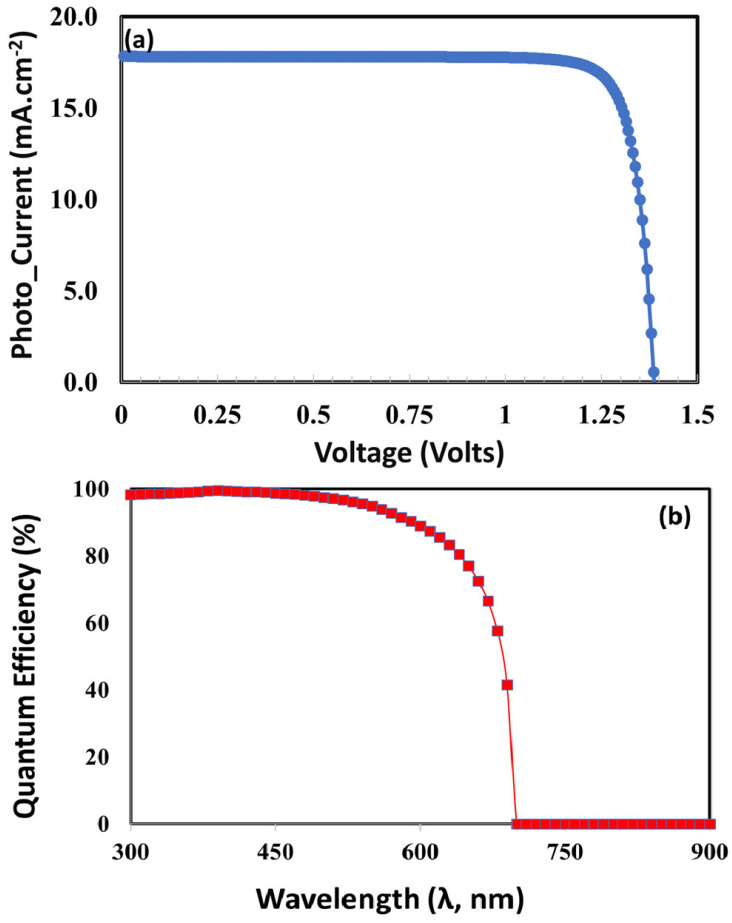
(**a**) The ITO/ETL/CaV_0.5_Fe_0.5_O_3_/Spiro-OMeTAD perovskite solar cell’s current density–voltage (J–V) characteristics under simulated AM 1.5G illumination. (**b**) The ITO/ETL/CaV_0.5_Fe_0.5_O_3_/Spiro-OMeTAD perovskite solar cell’s external quantum efficiency (EQE) spectrum. The plot displays the wavelength-dependent fraction of incident photons that are converted into electrons and collected at the external circuit.

Figure 7a shows the photocurrent voltage response of the proposed and optimized device. The optimized ITO/TiO_2_/CaV_0.5_Fe_0.5_O_3_/Cu_2_O perovskite solar cell demonstrates exceptional photovoltaic performance, attaining a power conversion efficiency of 23.28%, an open-circuit voltage of 1.38 V, a short-circuit current density of 19.8 mA/cm^2^, and a fill factor of 85.2%, respectively.

The quantum efficiency (QE) curve shown in Figure 7b for the highly optimized ITO/TiO_2_/CaV_0.5_Fe_0.5_O_3_/Cu_2_O device displays an efficient photon-to-electron conversion throughout a wide spectrum. The figure displays a pronounced and level plateau, sustaining over 80% efficiency from around 450 nm to 750 nm, indicating that the device effectively captures the bulk of photo-generated charge carriers over the visible light spectrum [74]. This exceptional performance stems from the optimized layer thicknesses and doping densities, which enhance light absorption in the 550 nm thick perovskite absorber and enable nearly lossless charge extraction at both interfaces, attributed to the highly doped and well-matched TiO_2_ electron transport layer and Cu_2_O hole transport layer. The pronounced decrease in quantum efficiency at the long-wavelength boundary (approximately 800 nm) accurately delineates the absorber’s bandgap, whereas the moderate reduction in the ultraviolet spectrum is due to parasitic absorption in the TiO_2_ layer, collectively affirming a high-quality, well-engineered photovoltaic device.

### 3.8. Interface Trapped Density Responses Between Perovskite and Transport Layer

**Figure 8:**
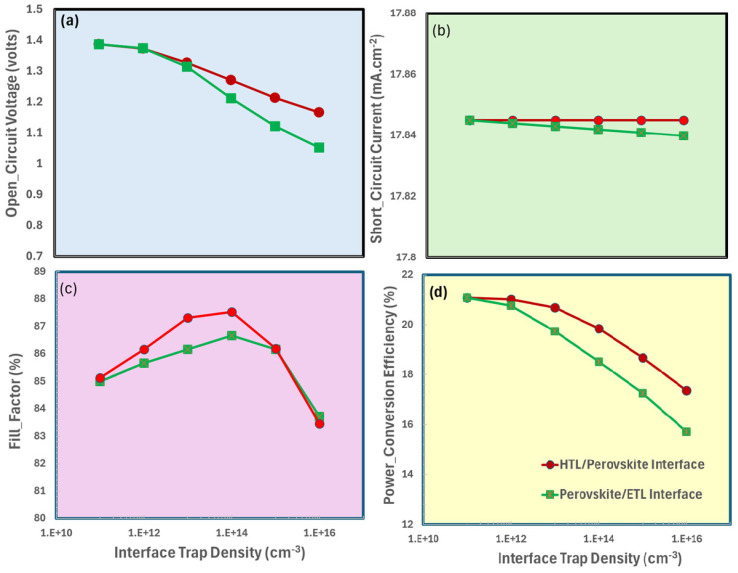
Photovoltaic performance parameters of ITO/TiO_2_/CaV_0.5_Fe_0.5_O_3_/Cu_2_O as a function of interface trap density: (**a**) open-circuit voltage (V_oc_), (**b**) short-circuit current density (J_sc_), (**c**) fill factor (FF), and (**d**) power conversion efficiency (PCE).

Figure 8a–d highlight the photovoltaic performance parameters of an ITO/TiO_2_/CaV_0.5_Fe_0.5_O_3_ perovskite solar cell, which are characterized by the interface trap density located between the ETL/perovskite layer and perovskite layer/HTL. These parameters include (i) open-circuit voltage, (ii) short-circuit current, (iii) fill-factor, and (iv) power-conversion efficiency. The open-circuit voltage decreases as both the HTL/Perovskite and Perovskite/ETL interface defects increase, as illustrated in Figure 8a. At higher interface trap densities, enhanced SRH trap-assisted recombination occurs, resulting in a reduction in carrier lifetime and quasi-Fermi level splitting [75,76,77]. Nonetheless, the decline is less pronounced for HTL/Perovskite defects, indicating that traps on the ETL side are more detrimental. Generation rate and optical absorption remain unchanged at higher trap density, resulting in a nearly constant short-circuit current density (see Figure 8b) of approximately 17.84 mA/cm^2^ in Figure 8b, irrespective of the interface trap density. The fill factor (see Figure 8c) rises from 10^10^ to around 10^14^ cm^−3^ due to improved charge extraction and reduced series resistance; however, it experiences a significant decline from 10^14^ to around 10^16^ cm^−3^ due to heightened recombination and resistive losses. FF is consistently elevated for HTL/Perovskite defects. The power conversion efficiency in Figure 8d is 21% at low doping but decreases significantly at 10^12^ cm^−3^, which can be attributed to reductions in V_oc_ and FF. The HTL/Perovskite defects demonstrate superior performance compared to ETL-side traps. Both interfaces incur losses due to trapping; however, the defects on the ETL side have a more significant negative impact on V_oc_ and PCE. The optimal doping density range is 10^12^–10^14^ cm^−3^, within which the fill factor is maximized, the open-circuit voltage remains near its peak, and the power conversion efficiency is significantly high.

### 3.9. Temperature Effects on Proposed Perovskite Solar Cell

Despite their high efficiency, perovskite solar cells face significant challenges when subjected to elevated temperatures. Numerous degradation processes affect these devices’ temperature sensitivity, thereby lowering their efficiency and stability with time. Power conversion efficiency markedly declines at elevated temperatures due to enhanced non-radiative recombination and worse charge extraction resulting from accelerated ion mobility inside the perovskite lattice, which contribute to defect formation and phase segregation. The evaluation of performance is further complicated by the worsening of current-voltage hysteresis due to the thermal activation of ionic species. Temperature-induced halide segregation promotes the degradation of mixed-halide perovskites by compromising their precisely designed bandgap frameworks [78,79,80].

**Figure 9:**
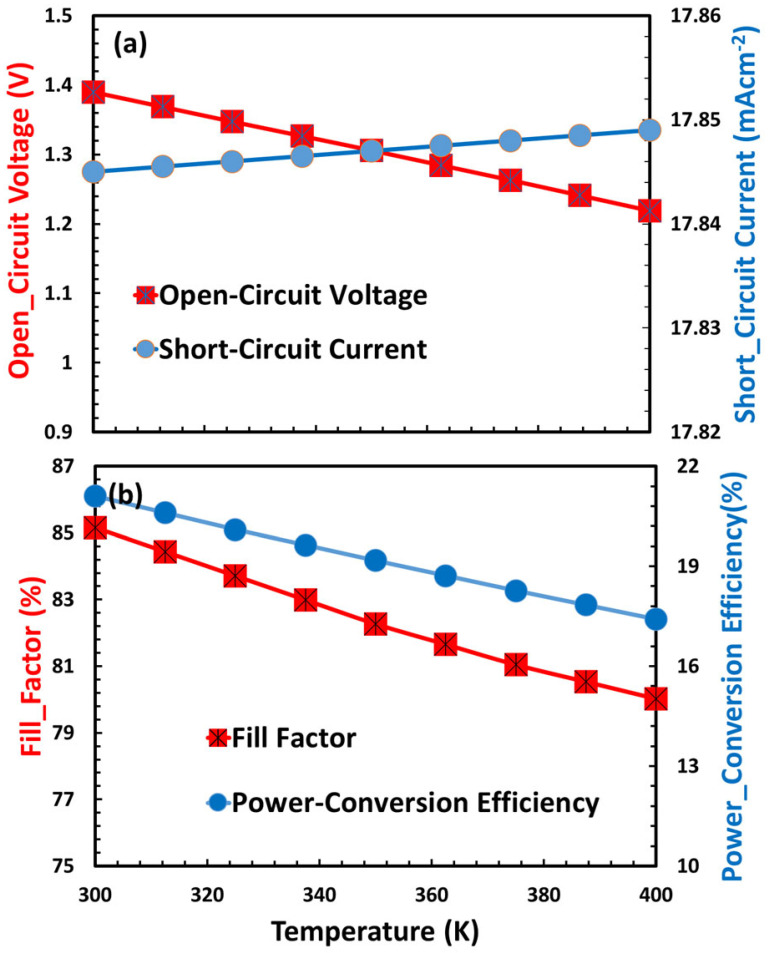
Photovoltaic performance parameters of ITO/TiO_2_/CaV_0.5_Fe_0.5_O_3_/Cu_2_O as a function of ambient temperature.

Temperature has a major impact on the performance of ITO/TiO_2_/CaV_0.5_Fe_0.5_O_3_/Cu_2_O perovskite solar cells, with important photovoltaic parameters showing clear thermal dependencies as shown in Figure 9a,b. Open-circuit voltage declines with rising temperature, which can be attributed to heightened intrinsic carrier concentration, bandgap narrowing, and increased non-radiative recombination, especially within the CaV_0.5_Fe_0.5_O_3_ active layer and at the TiO_2_/Cu_2_O heterojunction interface. The short-circuit current may exhibit a minor increase or stabilize, as elevated temperatures enhance charge mobility in TiO_2_ and Cu_2_O; however, parasitic absorption and defect formation may counteract these improvements. The fill factor typically decreases as a result of increasing series resistance, shunt pathways caused by ion migration, and interfacial degradation, which are further intensified by thermal expansion mismatches among layers.

As a result, the power conversion efficiency declines with increasing temperature, while the aggregate losses in V_oc_ FF, and potential J_sc_ saturation surpass any advantages in mobility. Some problems that only happen with the given materials are the oxidation of Cu_2_O, the instability of the phase CaV_0.5_Fe_0.5_O_0.3_, and the mixing of ITO and TiO_2_ at high temperatures.

### 3.10. Series Resistance and Shunt Resistance Effects on Proposed Perovskite Solar Cell

**Figure 10:**
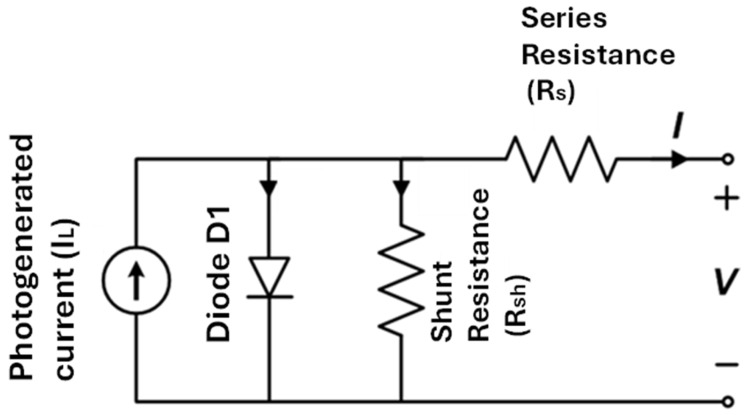
The equivalent circuit of a photovoltaic solar cell shown by a single-diode model. The model comprises a current source (I_L_) indicative of light-generated current, a diode (D1) simulating the rectifying behavior of the p-n junction, a series resistance (R_s_) that addresses resistive losses, and a shunt resistance (R_sh_) that signifies leakage current channels.

The equivalent circuit model is the most effective method for describing and evaluating the electrical properties of solar cells. The basic equivalent circuit, which employs an independent photogenerated optical absorption current source, is depicted in Figure 10. Recombination losses are represented as diode that is connected in parallel with the current source, while R_s_ and R_sh_ denote series and shunt configurations [81,82], respectively. Series resistance is generated by the resistive losses of the electrode, charge transport layer, and interfacial contact. Shunt resistance and leakage are induced by pinholes, particle boundaries, and active layer defects. The current loss is amplified by the reduced R_sh_ value, which in turn reduces V_oc_ and FF. We can improve the efficiency of solar cells by reducing the series resistance (R_s_) through the use of conductive electrodes and charge transport layers and by increasing the shunt resistance (R_sh_) through the use of high-quality coatings and defect passivation.

**Figure 11:**
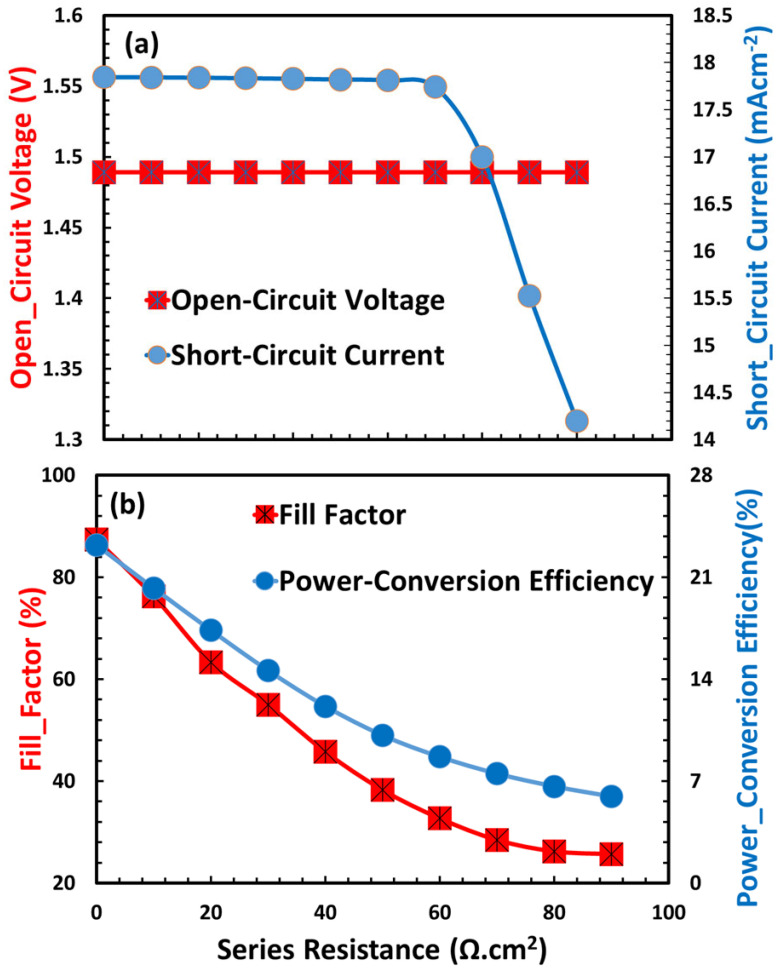
Impact of series resistance on the photovoltaic characteristics of a TiO_2_/CaV_0.5_Fe_0.5_O_3_/Cu_2_O solar cell.

Figure 11a,b shows the outcomes of an investigation regarding the impact of series resistance (R_s_) on the photovoltaic efficiency of the TiO_2_/CaV_0.5_Fe_0.5_/Cu_2_O device configuration. The figure demonstrates that a decrease in series resistance is essential for achieving maximum efficiency [83,84]. The rise in series resistance led to a decrease in short-circuit current, signifying increased charge carrier recombination and heightened parasitic resistive losses inside the solar cell. A significant reduction was seen in both the fill factor and the overall power-conversion efficiency when the series resistance went beyond its optimal limit. This trend highlights the critical need for improved charge transport layers and low-resistance interfaces in the device design to optimize the extraction of photogenerated carriers and minimize series resistance.

### 3.11. Shunt Resistance Effects on Proposed Perovskite Solar Cell

**Figure 12:**
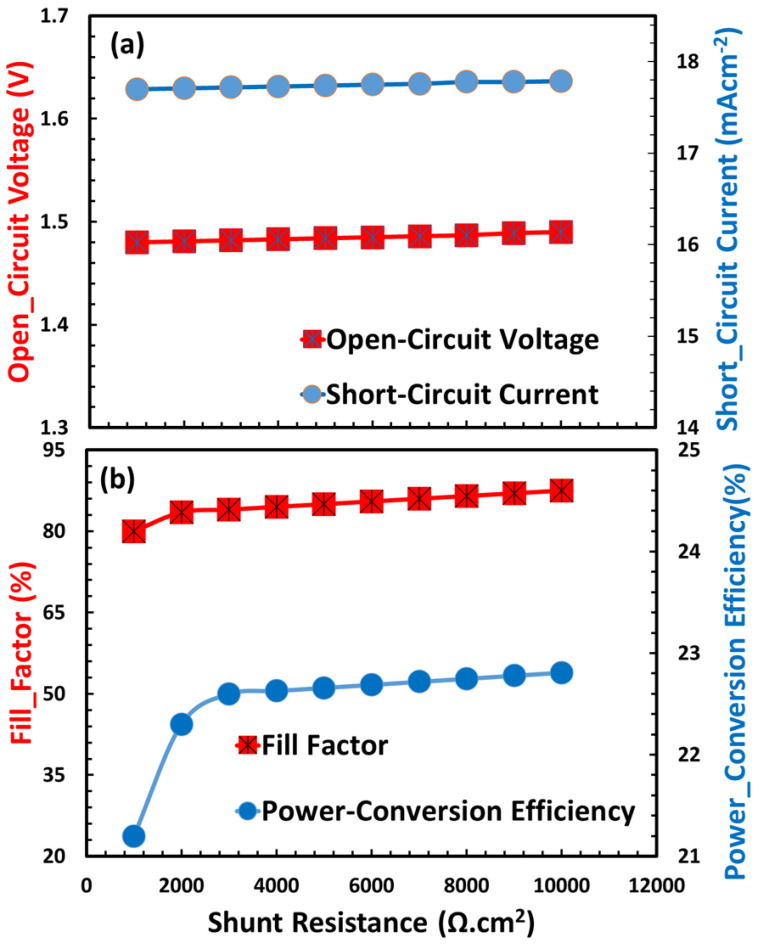
Impact of shunt resistance on the photovoltaic characteristics of a TiO_2_/CaV_0.5_Fe_0.5_O_3_/Cu_2_O solar cell: (**a**) open-circuit voltage and short-circuit current, (**b**) fill factor and power conversion efficiency.

Figure 12 shows the outcomes of this study regarding the impact of shunt resistance (R_sh_) on the photovoltaic efficiency of the TiO_2_/CaV_0.5_Fe_0.5_/Cu_2_O device configuration. The shunt resistance is a vital metric influencing the performance of the TiO_2_/CaV_0.5_Fe_0.5_O_3_/Cu_2_O solar cell, since it measures the extent of current leakage pathways inside the device. A high shunt resistance signifies a superior device with few parasitic losses. The influence on the essential photovoltaic parameters is significant and interrelated [85]. The open-circuit voltage is least sensitive to shunt resistance; as shown in Figure 12a, it nearly remains constant. The short-circuit current is initially not very (nearly negligibly) influenced by it, since the internal field effectively directs carriers to the contacts at zero bias. Nevertheless, if shunt resistance decreases enough, a considerable fraction of the photogenerated current is diverted along these parallel pathways, resulting in a quantifiable reduction in short circuit current. The fill factor and power conversion efficiency significantly decline due to a decreased shunt resistance, as shown in Figure 12b. A low shunt resistance results in a “soft” or rounded knee in the current–voltage characteristic curve, significantly diminishing the fill factor by constraining the maximum power point [86]. The total efficiency experiences a significant decline due to the combined deterioration of open-circuit voltage, short-circuit current and fill-factor, particularly the latter two parameters. Consequently, maximizing shunt resistance is essential for enhancing the performance of this heterojunction solar cell, since it directly mitigates harmful recombination and current leakage.

### 3.12. Photovoltaic Comparison of the Proposed Perovskite Solar Cell

Among the examined Fe-based perovskite solar cells, as shown in Table 4, the recently reported CaV_0.5_Fe_0.5_O_3_ exhibits remarkable photovoltaic performance, achieving an outstanding power-conversion efficiency of 23.28% within this material category. This exceptional outcome is attributed to its substantially elevated open-circuit voltage (1.38 V), an excellent fill factor (85.2%), and a robust short-circuit current density (19.8 mA/cm^2^). Thus, CaV_0.5_Fe_0.5_O_3_ markedly surpasses all previous documented iron-based perovskite oxides and competes with the performance of leading lead-based, Fe-doped MAI cells, establishing it as a very attractive candidate for next-generation photovoltaics.

## 4. Conclusions

The lead-free perovskite CaV_0.5_Fe_0.5_O_3_ has tremendous prospects as a stable, non-toxic, and high-performance absorber for next-generation solar cells, as this study convincingly illustrates. A record power conversion efficiency of 23.28% for a lead-free perovskite solar cell was achieved by developing an optimized device design, ITO/TiO_2_/CaV_0.5_FE_0.5_O_3_/Cu_2_O, using rigorous numerical modeling and transport layer engineering. An outstanding open-circuit voltage of 1.38 V, a short-circuit current density of 19.8 mA/cm^2^, and a fill factor of 85.2% define this achievement. The optimal choice and doping of TiO_2_ for the electron transport layer and Cu_2_O for the hole transport layer, because of their better band alignment, charge mobility, and stability, were important contributors in this success. Defects at the ETL/perovskite interface are more harmful than those at the HTL side, according to interface analysis, whereas the ideal balance between light absorption and charge extraction was achieved by identifying a 550 nm CaV_0.5_Fe_0.5_O_3_ absorber thickness. To maintain its excellent performance, the optimized device needed low series resistance (R_s_ < 1 Ω·cm^2^) and high shunt resistance (R_sh_ > 10^4^ Ω·cm^2^). It also showed strong thermal stability up to 350 K.

These results clearly demonstrate that CaV_0.5_Fe_0.5_O_3_ is a very practical and environmentally beneficial substitute for lead-based perovskites. Together with improved stability and non-toxicity, the attained efficiency of 23.28% makes CaV_0.5_Fe_0.5_O_3_-based solar cells a viable option for high-performance, scalable, and sustainable photovoltaics. A major step towards the commercialization of lead-free perovskite solar technology, this study offers a thorough roadmap for optimizing charge transport layers and interface features.

## Figures and Tables

**Table 1:** Listed the essential electronic and transport characteristics for the electron transport layers (ETLs: SnO_2_, ZnO, TiO_2_) and the perovskite absorber (CaV_0.5_Fe_0.5_O_3_) layer.

Photovoltaic Parameters	Unit	Symbol	SnO_2_	ZnO	TiO_2_	CaV_0.5_Fe_0.5_O_3_
Thickness	nm	Th	350	350	350	350
Energy Band Gap	eV	Eg	3.5	2.2	3.3	1.78
Electron Affinity	eV	χ	4.2	4.5	4	3.9
Dielectric Permittivity (Relative)		ε	9	9	10	19
Effective Density of States at Conduction Band	cm^−3^	N_C_	2.2 × 10^18^	2.2 × 10^18^	2.2 × 10^18^	1 × 10^19^
Effective Density of States at Valence Band	cm^−3^	N_V_	1.8 × 10^19^	1.8 × 10^19^	1.8 × 10^19^	1 × 10^19^
Hole Thermal Velocity	cm/s	V_h_	1 × 10^7^	1 × 10^7^	1 × 10^7^	1 × 10^7^
Electron Thermal Velocity	cm/s	Ve	1 × 10^7^	1 × 10^7^	1 × 10^7^	1 × 10^7^
Electron Mobility	cm^−2^/V·s	µ_e_	20	100	400	20
Hole Mobility	cm^−2^/V·s	µ_h_	10	25	110	10
Uniform Shallow Donor Doping	cm^−3^	N_D_	1 × 10^17^	1 × 10^17^	1 × 10^17^	1 × 10^17^
Uniform Shallow Acceptor Doping	cm^−3^	N_A_	0	0	0	0
Defect Density	cm^−3^	N_T_	1 × 10^15^	1 × 10^15^	1 × 10^15^	1 × 10^15^

**Table 2:** List of the essential electronic and transport characteristics for the hole transport layers including PEDOT:PSS, Spiro-OMeTAD, and Cu_2_O layer.

Photovoltaic Parameters	Unit	Symbol	PEDOT:PSS	Spiro OMeTAD	Cu_2_O
Thickness	nm	Th	350	350	350
Energy Band Gap	eV	Eg	2.2	2.9	2.17
Electron Affinity	eV	χ	2.9	2.2	3.2
Dielectric Permittivity		ε	3	3.5	7.11
Effective Density of States at Con-duction Band	cm^−3^	N_C_	2.2 × 10^18^	2.2 × 10^18^	2.5 × 10^17^
Effective Density of States at Valence Band	cm^−3^	N_V_	1.8 × 10^19^	1.8 × 10^18^	2.5 × 10^19^
Hole Thermal Velocity	cm/s	V_h_	1 × 10^7^	1 × 10^7^	1 × 10^7^
Electron Thermal Velocity	cm/s	Ve	1 × 10^7^	1 × 10^7^	1 × 10^7^
Electron Mobility	cm^−2^/V·s	µ_e_	10	1 × 10^−4^	200.00
Hole Mobility	cm^−2^/V·s	µ_h_	10	1 × 10^−1^	80.00
Uniform Shallow Donor Doping	cm^−3^	N_D_	0	0	0
Uniform Shallow Acceptor Doping	cm^−3^	N_A_	1 × 10^17^	1 × 10^17^	1 × 10^17^
Defect Density	cm^−3^	N_T_	1 × 10^15^	1 × 10^15^	1 × 10^15^
References			[18,51,55,56]	[57,58,59]	[60,61,62]

**Table 3:** Performance indicators of the completely optimized n-i-p structured TiO_2_/CaV_0.5_Fe_0.5_O_3_/Cu_2_O perovskite solar cell. The device attains a power conversion efficiency of 23.28% with optimized layer thicknesses and doping densities.

Transport Layer	Optimization Parameters		Unit	V_oc_ (V)	J_SC_ mA·cm^−2^	FF (%)	PCE (%)
Electron Transport Layer (TiO_2_)	Thickness	40	nm	1.38	19.8	85.2	23.28
	Doping Density	1 × 10^20^	cm^−3^				
Hole Transport Layer (Cu_2_O)	Thickness	50	nm				
	Doping Density	1 × 10^20^	cm^−3^				
Perovskite Absorber Layer CaV_0.5_Fe_0.5_O_3_	Thickness	550	nm				
	Doping Density	-	cm^−3^				

**Table 4:** Comparison of the photovoltaic performance of Fe-based perovskite solar cells with CaV_0.5_Fe_0.5_O_3_ material compositions for this investigation.

Perovskite Absorber	Hole Transport Layer	Electron Transport Layer	Open-Circuit Voltage	Short-Circuit Current	Fill-Factor	Power-Conversion Efficiency	Reference
MAI	Spiro OMeTAD	TiO_2_	1.07	20.19	67.92	14.67	[87]
(MAI)_0.991_(FeCl_2_)_0.009_	Spiro OMeTAD	TiO_2_	1.08	21.91	69.12	16.35	[87]
(MAI)_0.982_(FeCl_2_)_0.018_	Spiro OMeTAD	TiO_2_	1.1	22.21	70.85	17.31	[87]
(MAI)_0.964_(FeCl_2_)_0.036_	Spiro OMeTAD	TiO_2_	1.08	21.34	69.02	15.91	[87]
(MAI)_0.928_(FeCl_2_)_0.072_	Spiro OMeTAD	TiO_2_	1.08	18.94	65.14	13.32	[87]
Bi_0.8_La_0.2_FeO_3_	QSPE	TiO_2_	0.604	0.346	63.6	0.133	[88]
BiFeO	Graphite	ZnO	0.642	12.47	50.4	3.98	[89]
Doped-BiFeO_3_	NiO	TiO_2_	1.83	9.45	86.74	15	[90]
La_2_Ni_0.8_Fe_0.2_MnO_6_	Spiro OMeTAD	TiO_2_	0.495	1.6	47	0.37	[19]
La_2_Ni_0.8_Fe_0.2_MnO_6_	Spiro OMeTAD	ZrO_2_	0.74	8.5	58	3.7	[19]
CaV_0.5_Fe_0.5_O_3_	Spiro OMeTAD	TiO_2_	1.38	19.8	85.2	23.28	This Study

## Data Availability

Data is available on request.
